# Impact of Clinician-Supported Peer Health Navigation on Hospital Resource Utilisation amongst High Risk Adults: A Pragmatic Propensity-Score Matching Study

**DOI:** 10.5334/ijic.9105

**Published:** 2026-02-04

**Authors:** Rebecca L. Jessup, Keith Stockman, Cilla Haywood, Daniel Nguyen, Mark Tacey, Sarah Thomas, Donald Campbell

**Affiliations:** 1 Victorian Centre for Virtual Health Research, Northern Health, Epping, Australia; 2 School of Allied Health, Human Services and Sport, La Trobe University, Bundoora, Australia; 3 School of Rural Health, Monash University, Warragul, Australia; 4 Staying Well and Hospital Without Walls Program, Northern Health, Epping, Australia; 5 Faculty of Art, Design and Architecture, Monash University, Clayton, Victoria, Australia; 6 Austin Health Department of Medicine, Austin Health, Heidelberg, Australia; 7 Department of Medicine, University of Melbourne, Parkville, Australia

**Keywords:** peer health navigators, hospital admissions, emergency department presentations, integrated care, propensity score matching, outpatient non-attendance, resource utilisation

## Abstract

**Introduction:**

The Northern Patient Watch (NPW) program aimed to reduce hospital resource use by providing proactive support through peer health navigators working alongside health professionals. This study assessed the impact of NPW on hospital admissions, bed-days, emergency department presentations, and outpatient non-attendance rates, compared to propensity score-matched controls.

**Research Method:**

A propensity score matching design compared NPW enrolees with controls over 3-, 6-, and 12-month follow-up periods. Hospital resource utilisation was the primary outcome, with secondary outcomes including outpatient appointment non-attendance rates. Statistical methods addressed both normally and non-normally distributed variables.

**Results:**

NPW enrolees used fewer hospital bed-days at all time points compared to matched controls, with the greatest effect at 12 months (median 2.00 [CI 0.00, 8.00] vs. 4.00 [CI 1.00, 14.00]). Admissions were significantly lower at all time points, halving at 12 months (median 1.00 [CI 0.00, 4.00] vs. 2.00 [CI 1.00, 4.00]). Emergency presentations were lower in the NPW group but not statistically significant. Outpatient non-attendance rates were significantly reduced (12 months: 44.8% vs. 55.6%), showing improved healthcare engagement.

**Conclusion:**

The NPW programme reduced admissions, bed-days, and outpatient non-attendance, suggesting peer health navigators supported by health professionals improve resource use and patient engagement.

## Background

Health systems worldwide are facing increasing demand due to aging populations and increasing rates of chronic and complex diseases, with most health systems predicted to be unsustainable in their current form by 2050 [1,2,3]. These challenges are compounded by difficulties in retaining skilled healthcare professionals in the workforce who often experience high levels of burnout due to heavy workloads, staff shortages, and expectations to pivot toward rapidly evolving models of care [4,5]. As a result, there is a growing need for innovative strategies to manage the most resource-intensive patient groups, with research demonstrating that a small number of patients with chronic and complex care needs account for a disproportionately larger share of healthcare utilisation [6]. Identifying these individuals, through risk algorithms, presents an opportunity for targeted intervention to reduce preventable inpatient bed-based resource use [7]. One such approach involves incorporating lay health workers, such as patient or peer health navigators, into the healthcare workforce to develop risk-targeted, community-based models of care capable of addressing these systemic challenges.

Peer health navigators are individuals who assist patients in navigating healthcare systems. They provide guidance, emotional support, and information on available services, and help bridge gaps in care by addressing barriers to healthcare access and promoting better health outcomes [8]. Because they often share similar cultural backgrounds, health or social challenges with the communities they serve, they are well-positioned to build trust and encourage more effective healthcare engagement [9].

The roles performed by peer health navigators vary depending on their skills and the needs they address. Typically these roles include connecting patients and communities with reliable health information for self-management, offering more comprehensive, patient-centred care focused on individual needs rather than specific health conditions, identifying and addressing barriers to care, and linking people with relevant health and social services [8,10]. International studies demonstrate that peer health navigators improve access to care and prompt treatment initiation, increase adherence to treatment plans, boost attendance at both initial and follow-up appointments, and enhance referrals and continuity of care [11,12,13]. Additionally, they promote health system engagement, improve uptake of preventative health behaviours including vaccination and screening, and help reduce hospitalisations, 30-day readmissions, and emergency visits [13,14]. Peer health navigators work across various areas, including specific diseases (such as cancer, HIV, cardiovascular disease, and mental health) and settings (such as emergency departments and primary healthcare), or they may have a broader focus on navigation of systems and services [15]. A key factor in their success is the shared culture, language, and experiences they often have with the people they assist, which enhances healthcare engagement and improves patient outcomes [10,12,16,36].

In 2022, the World Health Organisation published a policy brief on patient navigators where they identified that there is good international evidence for these roles, and that they are particularly important for overcoming health equity gaps for underserved communities [17]. However, the brief also identified that the roles are context specific and so it can be hard to translate findings across countries and settings. This is particularly relevant in Australia, where unique factors such as geographical vastness, a highly diverse migrant population, diverse healthcare systems and variable access to ambulatory care, and the growing burden of multiple chronic diseases create specific challenges in delivering equitable, timely accessible care. The primary aim of this study was to determine the impact of a program incorporating peer health navigators working in a team with health qualified professionals targeting frequent hospital users at high risk of subsequent readmission on hospital resource use.

## Research Method

### Design

We used a propensity score matching design to compare hospital resource use for patients enrolled in a peer navigator program compared to nearest neighbours matched from those who declined to participate.

### Ethics

This study received ethical approval through the Northern Health Human Research Ethics Committee (HREC/69129/NH-2021-246618).

### Outcomes

The primary outcome for this study was hospital resource use, measured as hospital admissions, hospital bed days, emergency presentations and outpatient appointments.

### Setting and context

Northern Health (NH) serves as the primary provider of acute care in Melbourne’s outer northern metropolitan area. The area’s residents are culturally and linguistically diverse, with over 185 languages spoken [18]. The region has lower levels of income, educational achievement, and health literacy, alongside higher unemployment rates than the Victorian average [19]. An algorithm generated from administrative data is used to identify a cohort of inpatients who are a high risk of frequent return and subsequent admission, who constitute a focus for targeted intervention beyond conventional diseased focussed programs [7].

### Intervention

**Figure 1:**
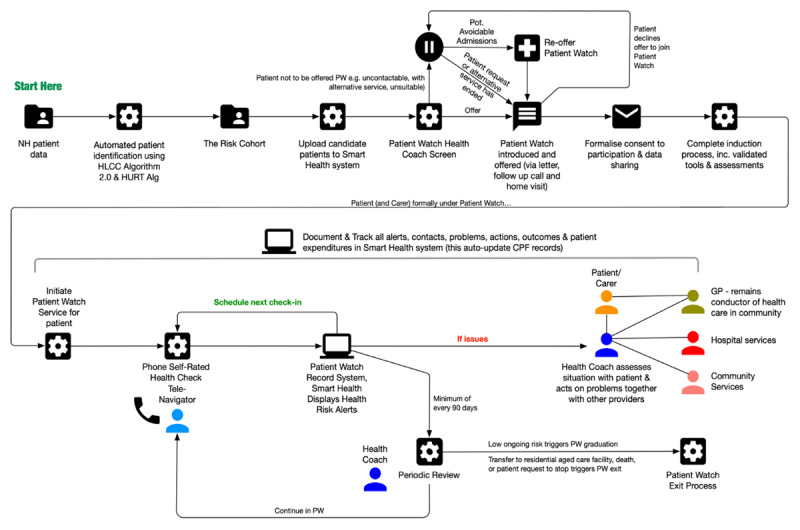
Patient watch model.

The intervention was Northern Patient Watch (NPW) (Figure 1), a model of care that involved monitoring at regular intervals (typically weekly), conducted by a peer health navigator (‘telecare-navigator’). Peer health navigators were recruited for their lived experience and their interpersonal strengths, based on research we completed to identify the skills and abilities required for the role. This included empathy, listening skills, and the ability to build trust with patients who are often socially or clinically vulnerable [20]. Formal training programs were not available in Australia at the time of the study, however navigators completed structured induction and training in communication, cultural safety, and escalation protocols, aligned with similar programs nationally [21]. They were salaried employees of the health service, with roles embedded in governance and supervision frameworks. This combination of lived experience, relational skill, and structured oversight ensured that navigators could effectively bridge communication and coordination gaps between patients, health professionals, and services.

The telecare-navigators monitored the health state of enrollees for a minimum of three months, potentially extending to years, using a combination of structured patient self-rated health checks by telephone. Telecare-navigators were supervised and supported by health professionals (‘health coaches’). Telephone monitoring was supported by a proprietary contact management, decision support and problem/action tracking application called Smart Health and Patient Journey Record [PaJR]) [22]. During business hours, participating patients or their carers could call NPW directly to ask questions or request assistance. NPW was not an emergency service; enrollees were educated regarding this and are provided with information about appropriate emergency pathways.

In response to signs of possible health decline, or other issues detected during the monitoring process, health coaches provided both reactive and proactive care and support. This included deliberate person-centred organisation of patient care activities between providers to facilitate self-management, and reducing barriers (e.g. transport or respite for dependents) to improve timeliness and access to appropriate care when required. Patients were encouraged by the health coaches to self-manage, understand their illness, and seek additional support and intervention when required. Health coaches worked with a wide array of clinicians and programs as well as external services providers, as required by the patient. The patients’ general practitioner remains the conductor of care in the community.

### Population

Patients were enrolled from November 2020. All eligible NPW patients who were screened by the 1^st^ April 2023 were included in the evaluation, to provide a 3-month period to start the program and provide a 12-month follow-up period to 30^th^ June 2024. Patients were selected for inclusion in the NPW using administrative dataset-based algorithms that aim to predict risk of future hospital admissions. The original algorithm was developed as part of the Health Links Chronic Care (HLCC) program, a Victorian Department of Health and Human Services patient identification and capitated funding initiative established with the support of the Australian Government Independent Health Pricing Authority This targeted those patients with chronic and complex care needs who were at high risk for three or more unplanned admissions in the subsequent 12 months [23]. The intent was to incentivise health services to better support these vulnerable patients, selected based on the risk-based selection algorithm, in the community and, as a consequence, reduce the number of potentially avoidable hospitalisations. The NPW program was set up as part of this program.

The HLCC-derived algorithm uses an unplanned index admission as a triggering event and then combines diagnostic information from that admission with demographic information to create a ‘risk score’ for the probability of three or more admissions in the next 12 months. Admission and demographic information include age, number of unplanned admissions in the past six months, emergency department visits in the past three months, hospitalisations due to certain progressive conditions and co-morbidities (such as asthma, kidney disease, COPD, heart disease, rheumatoid arthritis), smoking status, and type of residence (aged care or private residence). Patients that have an index admission and score above a threshold value determined by logistic regression analysis of historical data were eligible to be included in the PW program. This model has previously been found to have a non-significant impact in reducing unplanned readmissions to hospitals [23]. A model with higher sensitivity and specificity was subsequently developed by our team using administrative and clinical data (titled the Hospital Unplanned Readmissions Tool or HURT) [7], and a combination of both algorithms were used to identify eligible patients for NPW based on risk for subsequent admission on three or more occasions in the subsequent 12 months.

### Analysis

Propensity score matching (PSM) is an analytical method for studies that is designed to replicate the characteristics of a randomised controlled trial by using probability (or propensity) scores to ensure a balance in the observed baseline characteristics between a group that received treatment (NPW) and a group that did not (reference or control).

The initial sample size calculation was based on an expected reduction in bed day use per year of 25% as per the Monash Watch case study [24] that observed a reduction in the mean annual bed day use from 11.76 to 8.133). Thus, a sample size of 305 NPW cases was required with a 4:1 ratio between matched controls and cases (i.e. 1525 in total; 1 NPW case for 4 PSM controls) with 80% power to detect this change, and the probability of type 1 error being 0.05, assuming non-normal distribution of bed day use. This assumed that 10 patients were enrolled to NPW each week with recruitment commencing in November 2020.

The initial intention was to have the potential controls identified from all patients admitted to NH over the 1st July 2018 to November 2020 period, prior to the start of the NPW program, with up to 4 controls matched to each NPW program case. However, due to the known effect of the COVID-19 pandemic on the rate of emergency presentations and inpatient admissions for patients with chronic conditions (and specifically for those eligible for NPW [25]) and the difficulty in determining and accounting for the extent of this likely bias in the study, the methodology was subsequently changed to consider patients for the control group who were screened as eligible for enrolment in NPW and declined to participate. Whilst this methodology change aimed to minimise bias and determine the effectiveness of NPW, it has resulted in a reduced sample size, given only a 1:1 ratio between cases and controls can be obtained using the declined NPW patients as controls.

We applied nearest neighbour PSM for each NPW case, with Stata version 18.0 [26] *psmatch2* program utilised for this procedure to match the index episode for each NPW case. The index episode for each NPW case was considered to be the current inpatient episode when they qualified for either the HLCC or HURT algorithm, or the preceding episode if the patient met the criteria for inclusion in the NPW program whilst not currently admitted as an inpatient. Variables included in the PSM process included 5-year age category, sex, religion (broad groups according to ABS classifications), English-speaking, relationship status, admission type for index episode (i.e. emergency and elective), socio-economic status (SEIFA measure of economic advantage/disadvantage), discharge specialty, length of stay for index episode and Elixhauser score [27]. A look-back period of 3-years was used to identify Elixhauser comorbidities based on the International Classification of Diseases (ICD-10-AM) codes. Also included in the PSM process was prior healthcare utilisation in the 12-months preceding each eligible episode, including the number of emergency and elective admissions, total bed days, emergency presentations and outpatient appointments. As qualification for NPW was via identification using both the HLCC and/or HURT algorithm, this was also considered as part of the PSM process, with the combined algorithms increasing the specificity for detection of admission.

Outcomes were assessed from the date on which enrolment in the NPW program began for each NPW case, with the same time-gap between the index episode and this start date applied to each propensity matched control. Outcomes were assessed at three different endpoints: at 3-months, 6-months, and at 12-months follow-up. 12-months was considered to be the primary study period. Inpatient admissions and bed days were stratified by emergency and elective admission types. Patients were excluded from the analysis and comparison between cases and controls if either paired patient died in the period leading up to the specified follow-up timepoint.

### Statistical methods

Data were prepared in csv files by the Northern Health Decision Support Unit (DSU) with statistical analyses conducted using Stata version 18.0 (Stata Corp, College Station, Texas, USA). Variables that were normally distributed are presented as means and standard deviations with Student’s t-tests or Analysis of Variance (ANOVA) used to test for differences between groups. Skewed, non-normally distributed variables are presented as Median (Inter-quartile range – IQR), with Mann-Whitney (ranksum) tests used to test for group differences. Categorical variable differences were tested for using the chi-squared or Fishers Exact tests. A two-sided p-value of less than 0.05 will be used to indicate statistical significance. Medians and interquartile ranges were reported as primary measures, with means included descriptively.

## Results

As of 30^th^ June 2024, a total of 3,422 patients were identified by the algorithms as potential candidates for NPW, with 2,315 identified prior to the 1^st^ April 2023 to be included in the evaluation. A total of 930 were subsequently identified as unsuitable for the program (not available via telephone, significant cognitive impairment, involved in other appropriate support services) and 212 were pending or lost to follow-up after initial contact, leaving 1,173 who were contacted for enrolment. A total of 504 patients were initially enrolled and 320 completed or continued in the program at the time of study and were thus considered part of the NPW enrolee cohort for this study. There were 669 patients identified by either algorithm who declined to participate but were considered eligible candidates and were used as the matched control group. Table 1 provides an overview of the characteristics of the matched controls and enrollees. A small number of enrolled patients were not able to be matched as follows: no inpatient data to enable analysis to be conducted (n = 4); patients who are admitted 3 times per week for haemodialysis, which would impact outcome data (n = 22); non HLCC or HURT cases that were referred in from an alternative service (n = 20), and unable to identify a suitable match with PSM (n = 1).

**Table 1:** Characteristics of propensity score matched controls and Northern Patient Watch (NPW) enrolees.


CHARACTERISTICS	CONTROLS (n = 273)	NPW ENROLEES (n = 273)	p-VALUE

AGE			1.00

20–24	1 (0.4%)	2 (0.7%)	

30–34	5 (1.8%)	6 (2.2%)	

35–39	4 (1.5%)	4 (1.5%)	

40–44	5 (1.8%)	5 (1.8%)	

45–49	9 (3.3%)	7 (2.6%)	

50–54	14 (5.1%)	13 (4.8%)	

55–59	17 (6.2%)	16 (5.9%)	

60–64	31 (11.4%)	27 (9.9%)	

65–69	28 (10.3%)	34 (12.5%)	

70–74	43 (15.8%)	41 (15.0%)	

75–79	43 (15.8%)	52 (19.0%)	

80–84	39 (14.3%)	38 (13.9%)	

85–89	29 (10.6%)	23 (8.4%)	

90+	5 (1.8%)	5 (1.8%)	

GENDER			0.86

Male	121 (44.3%)	118 (43.2%)	

Female	152 (55.7%)	155 (56.8%)	

ABORIGINAL OR TORRES STRAIT ISLANDER			1.00

Yes	4 (1.5%)	4 (1.5%)	

RELIGION			0.64

Christianity	168 (61.5%)	181 (66.3%)	

Hinduism	2 (0.7%)	2 (0.7%)	

Islam	22 (8.1%)	21 (7.7%)	

Other Religions	2 (0.7%)	4 (1.5%)	

Secular/No Religion	79 (28.9%)	65 (23.8%)	

RELATIONSHIP STATUS			0.55

Married/De facto	144 (52.7%)	136 (49.8%)	

Single/Widowed/Divorced	129 (47.3%)	137 (50.2%)	

SEIFA SCORE*, mean (SD)	945.1 (60.5)	947.4 (60.2)	0.65

SEIFA LOWER QUARTILE	92 (33.7%)	92 (33.7%)	1.00

ADMISSION TYPE**			1.00

Emergency	230 (84.2%)	231 (84.6%)	

Elective	43 (15.8%)	42 (15.4%)	

OVERALL LENGTH OF STAY (days)**, median (IQR)	1.0 (1.0, 3.0)	1.0 (1.0, 4.0)	0.38

ENGLISH SPEAKING	209 (76.6%)	220 (80.6%)	0.25

REQUIRES AN INTERPRETER	49 (17.9%)	40 (14.7%)	0.30

ANY PRIOR CHEMOTHERAPY/RADIOTHERAPY	22 (8.1%)	26 (9.5%)	0.55

ELIXHAUSER SCORE, mean (SD)	8.9 (8.9)	9.2 (9.1)	0.72

HEALTH CONDITIONS			

Congestive heart failure	62 (22.7%)	71 (26.0%)	0.37

Cardiac arrhythmias	84 (30.8%)	94 (34.4%)	0.36

Valvular disease	7 (2.6%)	10 (3.7%)	0.46

Pulmonary circulation disorders	9 (3.3%)	16 (5.9%)	0.15

Peripheral vascular disorders	16 (5.9%)	15 (5.5%)	0.85

Hypertension	64 (23.4%)	65 (23.8%)	0.92

Paralysis	6 (2.2%)	7 (2.6%)	0.78

Other neurological disorders	19 (7.0%)	16 (5.9%)	0.60

Chronic pulmonary disease	69 (25.3%)	57 (20.9%)	0.22

Diabetes, uncomplicated	90 (33.0%)	94 (34.4%)	0.72

Diabetes, complicated	93 (34.1%)	97 (35.5%)	0.72

Hypothyroidism	11 (4.0%)	4 (1.5%)	0.067

Renal failure	32 (11.7%)	45 (16.5%)	0.11

Liver disease	23 (8.4%)	17 (6.2%)	0.32

Peptic Ulcer disease	3 (1.1%)	8 (2.9%)	0.13

Lymphoma	0 (0.0%)	0 (0.0%)	1.00

Metastatic cancer	2 (0.7%)	2 (0.7%)	1.00

Solid tumour without metastasis	9 (3.3%)	14 (5.1%)	0.29

Rheumatoid arthritis	9 (3.3%)	11 (4.0%)	0.65

Coagulopathy	10 (3.7%)	14 (5.1%)	0.40

Obesity	6 (2.2%)	5 (1.8%)	0.76

Weight loss	53 (19.4%)	44 (16.1%)	0.31

Fluid and electrolyte disorders	107 (39.2%)	110 (40.3%)	0.79

Blood loss anaemia	10 (3.7%)	5 (1.8%)	0.19

Deficiency anaemias	36 (13.2%)	48 (17.6%)	0.15

Alcohol abuse	14 (5.1%)	14 (5.1%)	1.00

Drug abuse	6 (2.2%)	6 (2.2%)	1.00

Psychoses	0 (0.0%)	2 (0.7%)	0.16

Depression	14 (5.1%)	13 (4.8%)	0.84

IN-HOSPITAL COMPLICATION**	24 (8.8%)	32 (11.7%)	0.26


*The SEIFA score (Socio-Economic Indexes for Areas) is a set of measures developed by the Australian Bureau of Statistics (ABS) to rank areas in Australia according to their relative socio-economic advantage and disadvantage. The scale used here was the IRSAD (Index of Relative Socio-economic Disadvantage) which ranges from 500–1200 with the average being 1000. The lower quartile indicates that the patient is from a postcode in the lowest 25% of the country. ** Admission type, in-hospital complication and length of stay for Index Admission.

A total of 273 pairs were matched. The propensity score matching process provided a reduction in the standardised mean difference from 6.26 (SD 6.79) to 3.20 (SD 2.94), with a Median of 2.6 (IQR: 0.15 – 5.13). The 95% percentile in the matched standardised differences was 8.43. Table 1 also demonstrates that the propensity score matching process provided a suitable match between the NPW cases and selected controls, given the limitations of the PSM process and the limited number of declined patients eligible to be considered as controls.

The comparison of NPW cases with matched controls over 3, 6, and 12-month follow-up periods demonstrates differences in hospital bed-day utilisation and outpatient attendance (Table 2). Results are presented as means (SD) and medians (IQR), with inpatient admission and bed-day data non-normally distributed. For admissions, NPW cases consistently had fewer admissions compared to controls across all time points (0.66 vs 0.89, p = 0.014; 6 months: 1.31 vs 1.88, p = 0.001; 12 months: 2.52 vs 3.11, p = 0.070). The differences were statistically significant at all time points when considering statistical tests corresponding to the non-normally distributed format of admissions.

**Table 2:** Hospital utilisation at 3-(n = 267), 6-(n = 260) and 12-months (n = 242)*.


OUTCOME		CONTROLS	NPW ENROLEES	p-VALUE

**Admissions**				

3 months	mean (SD)	0.89 (1.18)	0.66 (0.99)	**0.014**

	median (IQR)	1.00 (0.00, 1.00)	0.00 (0.00, 1.00)	**0.004**

6 months	mean (SD)	1.88 (2.32)	1.31 (1.67)	**0.001**

	median (IQR)	1.00 (0.00, 3.00)	1.00 (0.00, 2.00)	**<0.001**

12 months	mean (SD)	3.11 (4.03)	2.52 (3.06)	0.070

	median (IQR)	2.00 (1.00, 4.00)	1.00 (0.00, 4.00)	**0.007**

**Length of stay/bed-days**				

3 months	mean (SD)	5.88 (14.89)	2.27 (7.52)	**<0.001**

	median (IQR)	1.00 (0.00, 4.00)	0.00 (0.00, 1.00)	**<0.001**

6 months	mean (SD)	9.93 (19.50)	5.01 (13.28)	**<0.001**

	median (IQR)	2.00 (0.00, 9.00)	1.00 (0.00, 4.00)	**<0.001**

12 months	mean (SD)	14.08 (24.26)	9.48 (19.23)	0.021

	median (IQR)	4.00 (1.00, 15.00)	2.00 (0.00, 8.00)	**<0.001**

**Emergency Presentations**				

3 months	mean (SD)	0.21 (0.53)	0.21 (0.51)	0.93

	median (IQR)	0.00 (0.00, 0.00)	0.00 (0.00, 0.00)	0.63

6 months	mean (SD)	0.41 (0.69)	0.50 (0.84)	0.17

	median (IQR)	0.00 (0.00, 1.00)	0.00 (0.00, 1.00)	0.33

12 months	mean (SD)	0.77 (1.03)	0.81 (1.07)	0.73

	median (IQR)	0.00 (0.00, 1.00)	0.00 (0.00, 1.00)	0.78

**Any non-attendance at outpatient appointment(s)**				

3 months	n (%)	82 (30.7%)	50 (18.7%)	**0.001**

6 months	n (%)	115 (44.2%)	85 (32.7%)	**0.007**

12 months	n (%)	133 (55.0%)	108 (44.6%)	**0.023**


* Patient pair totals reduce over follow-up due to deaths of either the cases or controls in each pair. A pair is maintained throughout. NB: significant.

No statistically significant differences in emergency presentations were observed between the NPW and control groups (3 months: 0.21 vs 0.21, p = 0.93; and 12 months: 0.81 vs 0.77, p = 0.73). Any non-attendance at outpatient appointments were significantly lower for NPW cases at both 3 months (18.7% vs 30.7%, p = 0.001) and 12 months (44.6% vs 55.0%, p = 0.023).

**Figure 2:**
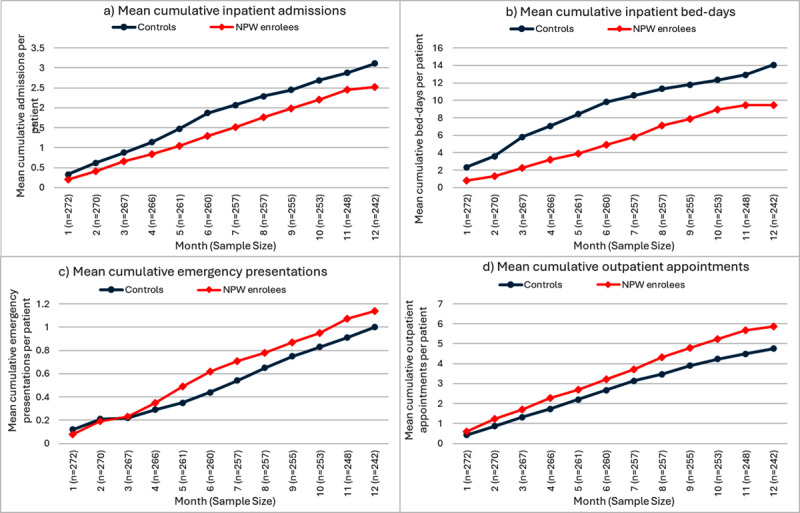
Cumulative inpatient admissions, bed-days, emergency presentations and outpatient appointments for NPW verses controls.

Figure 2 provides an illustration of the healthcare utilisation trends for NPW compared to controls over 12 months, demonstrating that the greatest relative effect size for length of stay was seen at months 5–7.

## Discussion

We evaluated the impact of a targeted peer health navigator programme, supported by health professionals, on hospital resource utilisation for patients identified as being at high risk of multiple subsequent hospital admissions. Overall, NPW enrolees had significantly fewer inpatient admissions and lower total bed-days during the 12-month follow up period following enrolment after an index admission than matched controls, with a growing effect over time, suggesting that the program’s impact strengthens as patients stabilise and remain enrolled for longer. While differences in emergency presentations were not statistically significant, NPW enrolees showed improved engagement with outpatient services, evidenced by lower non-attendance rates over time.

Our findings suggest that peer health navigators, supported by qualified health professionals, may assist patients to manage their health more effectively. By providing patients with guidance, emotional support, and practical assistance, peer health navigators may help patients engage and adhere to treatment plans more consistently, recognise early warning signs of deterioration, and seek timely medical advice. This ability to intervene early likely contributes to the reduction in prolonged hospital stays as evidenced by reduced hospital bed-day usage, even if it does not prevent emergency presentations altogether. However, by addressing issues before they escalate into more severe health crises, peer navigators may help minimise the duration of non-avoidable hospitalisations when they do occur as well as improving engagement with ambulatory care. Over time, the impact of the program may also grow as patients develop greater confidence and skills to self-manage their chronic conditions. Education, the adoption of a sense of agency, and physiological adaptation often require sustained effort and consistency to take full effect. As patients gradually internalise self-management strategies and experience greater control over their health, the benefits can compound, leading to improved health outcomes and reduced reliance on acute care services in the longer term. This highlights the importance of continuity and sustained support in complex chronic disease management, where even incremental gains can have an important cumulative effect over time.

Internationally, navigator programmes have been implemented in a variety of forms, ranging from volunteer or community-based initiatives to professionally trained and salaried roles embedded within healthcare organisations. In North America, for instance, navigators are widely used to support people with chronic and complex conditions across primary care, oncology, and mental health settings, with evidence of improved care coordination, adherence, and reduced unplanned admissions [8,28,29,30,31,32]. In contrast, many European systems integrate navigation within strong primary healthcare teams or post-discharge nurse-led models rather than through peer or lay roles. The NPW model represents a hybrid approach, combining the relational strengths of peer support with the structure and accountability of professional supervision, to deliver continuity of care across hospital and community interfaces.

Within Australia, health navigation programs remain relatively fragmented, often limited to disease-specific pilots or hospital-initiated outreach [9,37]. This study contributes to a growing body of evidence supporting system-level integration of navigators into mainstream care delivery. Its implementation within a public health service, targeting patients at high risk of hospital readmission, demonstrates feasibility and potential scalability. The model aligns closely with peak body recommendations around access to peer navigator support, and national primary healthcare reforms that emphasise integrated, multidisciplinary, person-centred care, particularly for populations experiencing health and social disadvantage [33,34].

Unlike a study in the US that observed declines in emergency presentations, NPW participants maintained similar ED attendance [35]. This may reflect Australia’s universal access to emergency care, differences in triage thresholds, or the program’s design focus on stabilisation rather than acute avoidance. Importantly, navigator roles are intended to completment rather than replaced existing process and systems, working alongside general practitioners, community health teams, hospital services and social care agencies to close the gaps in between-service coordination.

The findings from this study also provide context for the World Health Organization’s recommendation that patient and peer navigators can improve care integration and equity, especially for underserved populations [17]. While our results broadly support the conclusions made in this policy paper, they extend current understanding by demonstrating that navigator programs embedded within formal health service structures can also deliver measurable reductions in hospital utilisation. This suggests that WHO recommendations could be broadened to recognise the value of hybrid models where navigators function as part of multidisciplinary, clinically supervised teams.

The strengths of this study include the robust propensity score matching design, which minimised confounding variables and allowed for a more accurate comparison between NPW enrolees and controls. Additionally, the use of real-world hospital data over a prolonged period provides valuable insights into the effectiveness of the NPW program. However, attrition from deaths, withdrawals, and unmatched patients may have preferentially removed higher-risk individuals from later analyses, potentially attenuating true program effects or biasing findings toward lower utilisation. Similarly, the reliance on participants who declined the NPW programme as controls was a limitation and may have introduced some selection bias. The study also faced challenges in reaching the intended sample size, partly due to the impact of COVID-19 and the limited pool of suitable matches among patients who had declined the service. Finally, staff also reported that the reason many patients turned down the service was the presence of sufficient social support at home rather than due to poorer health status or disengagement, suggesting that any residual bias would likely reduce rather than exaggerate the observed program effect. Future research should consider a randomised controlled trial that ensures patient characteristics are the same at baseline, with consideration for collecting data on factors including availability of social support and loneliness as covariates to better assess its impact and includes a sensitivity analysis to determine which populations benefit most from the intervention. In addition, future studies should include information on the sociodemographic characteristics and lived experience of navigators to better understand how their backgrounds influence engagement and outcomes, as well as patient-reported measures such as quality of life to capture differences not reflected in hospital utilisation data.

Our study has several implications for the role of peer health navigators as part of frontline healthcare teams managing high admission risk patients in Australia. The results suggest that the incorporation of peer health navigators in teams may increase patient engagement, leading to significant resource savings, even after including the additional costs of the navigators as members of the health workforce. Further research should explore the long-term sustainability of such programmes and their potential impact on clinical outcomes across disparate groups of patients, particularly in diverse healthcare settings. Expanding the scope of future studies to include patient-reported outcomes could also provide a more comprehensive understanding of the programme’s impact on patient satisfaction and quality of life.

## Conclusion

The Northern Patient Watch (NPW) program highlights the measurable value of implementing a peer health navigator model of care within the health service. This model utilises an algorithm derived from administrative data sets to identify patients at high risk of hospital readmission after an initial admission. These identified patients benefit from a targeted, enduring relationship-based model of care, where appropriately trained and supervised peer health navigators, as frontline team members, collaborate closely with health professionals. The findings from this study suggest that this model can reduce unnecessary hospital bed day use and improve outpatient attendance. While there was no evidence of a reduction in emergency presentations, the overall trends indicate that peer health navigators may contribute to more efficient use of healthcare resources. Future research should incorporate a comprehensive health economic analysis from both hospital and patient perspectives to assess the long-term clinical and cost-effectiveness of these programs. It should also examine their wider impact on patient outcomes, especially across diverse healthcare settings. These models could play an important role in managing the increasing demands on healthcare systems by providing targeted, patient-centred care to support self-management and timely access to services. Funding and policy settings need to be adjusted in order to realise the value from such programs.

## Involvement of people with lived experience

This study was overseen by a steering committee with consumers with lived experience as members. This involvement has ensured that the Northern Patient Watch program itself, as well as the evaluation, aligned with the needs and priorities of patients. They provided input into the study design, offering insights to make protocols, recruitment strategies, and participant materials more inclusive and accessible. Consumers also guided the development of clear, participant-friendly communication and identified potential barriers to participation to the patient watch program to promote inclusivity (which will be reported in a subsequent paper). Throughout the study, they monitored progress to ensure it remained participant-centred and ethically sound, while also shaping the dissemination of findings to maximise community impact and practical application.
